# No Evidence for Threat-Induced Spatial Prioritization of Somatosensory Stimulation during Pain Control Using a Synchrony Judgment Paradigm

**DOI:** 10.1371/journal.pone.0156648

**Published:** 2016-06-07

**Authors:** Wouter Durnez, Stefaan Van Damme

**Affiliations:** Department of Experimental-Clinical and Health Psychology, Ghent University, Ghent, Belgium; University of Montreal, CANADA

## Abstract

Topical research efforts on attention to pain often take a critical look at the modulatory role of top-down factors. For instance, it has been shown that the fearful expectation of pain at a location of the body directs attention towards that body part. In addition, motivated attempts to control this pain were found to modulate this prioritization effect. Such studies have often used a temporal order judgment task, requiring participants to judge the order in which two stimuli are presented by indicating which one they perceived first. As this constitutes a forced-choice response format, such studies may be subject to response bias. The aim of the current study was to address this concern. We used a ternary synchrony judgment paradigm, in which participants judged the order in which two somatosensory stimuli occurred. Critically, participants now also had the option to give a ‘simultaneous’ response when they did not perceive a difference. This way we eliminated the need for guessing, and thus reduced the risk of response bias. One location was threatened with the possibility of pain in half of the trials, as predicted by an auditory cue. Additionally, half of the participants (pain control group) were encouraged to avoid pain stimuli by executing a quick button press. The other half (comparison group) performed a similar action, albeit unrelated to the occurrence of pain. Our data did not support threat-induced spatial prioritization, nor did we find evidence that pain control attempts influenced attention in any way.

## Introduction

In a recent surge of research endeavors, pain has been investigated as part of a motivational setting [1–3]. Indeed, aside from to its inherent capability of capturing attention in a bottom-up manner, it has long been clear that anticipating pain may trigger top-down modulation of attentional selection. More specifically, it is suggested that the anticipation of physical harm–naturally inciting basal goals of self-preservation–leads to preferential allocation of attentional resources to stimuli that share features with the expected pain stimulus [4].

Case in point, expecting pain to occur on a location of the body has been shown to direct attention towards that location. Data showed that the anticipation of pain at a specific body location caused tactile stimuli administered to that location to be perceived more easily [5,6] and quicker [7,8], compared to tactile input stemming from the other location. The value of pain control motivation–a second top-down factor in this narrative–was highlighted in a number of recent studies. For instance, it was shown that an attentional bias towards visual pain cues was enhanced when participants engaged in pain control behavior [9]. More recently, it was found that the expectation of pain directed attention towards a currently threatened location, but the inclusion of pain control attempts generalized this prioritization to safe situations [6]. Furthermore, encouraging participants to avoid pain was found to be a critical component of threat-induced attentional prioritization of somatosensory information, when presented in competition with visual input [10]. The sum of these results clearly underpins the value of modeling active goal pursuit in experimental settings.

A number of the aforementioned findings were obtained by means of a temporal order judgment (TOJ) paradigm. In this experimental setup, participants are requested to judge the order in which two stimuli were presented by stating which one they perceived first. One of the stimulus locations was threatened with a painful stimulus in half of all trials. While this approach often yields convincing results, the method itself is not without controversy. A possible criticism is that participants were forced to make a (binary) choice, with no possibility to report that they did not perceive a temporal difference. This leaves the paradigm vulnerable to response bias, which could potentially contaminate the performance measures used in hypothesis testing [11,12]. For instance, perceptually undecided participants could be inclined to respond with the pain location, due to its increased experimental salience. Performance measures could then show the impact of pain anticipation on the decision making process, rather than reflect an effect on somatosensory attention [13]. In contrast, synchrony judgment (SJ) formats allow participants to report the stimulus order as ‘simultaneous’ (an S response), effectively curbing the chances of measuring response bias. Consequently, SJ paradigms have been argued to be the superior choice when investigating the temporal perception of sensory events [11–15]. It would then be interesting to attempt to demonstrate spatial prioritization of a body location threatened with pain using the SJ response format instead.

In the current study, we set out to address this challenge. In a ternary SJ paradigm participants judged the order in which two somatosensory stimuli were presented–one on the right hand and one on the left hand. Apart from the response options ‘left first’ and ‘right first’, a third ‘simultaneous’ response could be given. In half of all trials one location was threatened with a painful stimulus. This was signaled by means of an auditory cue: one predictive of possible pain, the other foretelling a pain-free trial. Our first aim was to replicate the finding that the threat of pain can prioritize attention towards the threatened body site [5–8] (hypothesis 1). In addition, we sought to expand the experiment’s design by encouraging active pain control behavior in half of all participants. This pain control group was told they could avoid the potential administration of a painful stimulus by executing a quick button press. The comparison group, on the other hand, was given a similar assignment that was not related to pain avoidance. Based upon previous results [6,10], we expected pain control attempts to enhance spatial prioritization (hypothesis 2a), to generalize this prioritization to neutral trials (hypothesis 2b), or a combination of both.

## Method

### Participants

Forty students of Ghent University (9 male and 31 female; M_age_ 22 SD_age_ 2.86) participated in this study, either to earn required course credits or in exchange for a small financial compensation. Five of them were left-handed. All participants had normal or corrected-to-normal vision and normal hearing. The study protocol was approved by the Ethics Committee of the Faculty of Psychology and Educational Sciences of Ghent University. The experiment took approximately 1 hour and 10 minutes. All participants signed an informed consent form.

### Apparatus and stimulus material

The experiment was conducted in a darkened, sound isolated room. Participants sat on a chair in front a desk. Their hands were placed palm-down on marked positions. The tactile stimuli used in the experiment were vibrations, presented by means of two resonant-type tactors (C-2 TACTOR, Engineering Acoustics, Inc.) consisting of a housing of 3.05 cm diameter and 0.79 cm high, with a skin contactor of 0.76 cm diameter. Their functioning was controlled and amplified through a custom-built device. The tactors were attached directly to the skin in the center of the back of either hand using double-sided tape rings. The frequency of tactile stimulation was 200 Hz. The stimulus duration was set to 20 ms. In between both hands a red fixation LED (light-emitting diode) was placed, serving as a fixation point throughout the different trials of the experiment. Painful stimuli were generated electrically through means of constant current stimulators (Digitimer DS5, 2000). They were delivered via 2 lubricated Fukuda standard Ag/AgCl electrodes (1 cm diameter), placed in close proximity to the tactors and the superficial branch of the radial nerve. These sinusoid electrocutaneous stimuli had a frequency of 200 Hz and a duration of 200 ms. Amplitudes for both the tactile and electrocutaneous stimulation were set using adaptive procedures, as described in the procedure section. Auditory cues were administered using a set of headphones (Sennheiser HD 202 II). These cues consisted of either a high tone (1000 Hz) or a low tone (250 Hz). As part of the goal manipulation, participants were asked to press a foot pedal at specific moments in a portion of the trials. This foot pedal (M-Audio SP-1 sustain pedal) was attached to the floor at a distance that was comfortable for each participant, so that they could easily and quickly press down on it with their dominant foot. The pedal was connected to a Cedrus response box (RB-530 model) to optimize response time registration.

### SJ paradigm

The task was programmed in the programming language C using the Tscope 5 library package, an upgraded version of the original Tscope [16]. It ran on a laptop (Dell latitude E5520). Participants were instructed to keep their hands on the marked positions, and keep their gaze fixed on the fixation LED.

The experiment was divided into 4 blocks of 105 trials each, resulting in a total number of 420 trials. There were 2 blocks where the left hand was threatened by electrocutaneous stimulation, and 2 blocks where the right hand was threatened. Blocks were counterbalanced in this regard. Prior to each block, the experimenter informed the participants which hand was subject to possible painful stimulation. Additionally, they were given at least one electrocutaneous stimulus in the first ten trials, in order to re-establish contingency perception.

Each trial began with an illumination of the fixation LED for 1000 ms. Next, a 1000 ms auditory cue was presented, indicating whether or not an electrocutaneous stimulus could follow (within-subjects variable of THREAT). One tone frequency predicted the possible advent of such a stimulus (threat trial), while the other signaled that this would not be the case (neutral trial). The frequency of the threatening tone (high versus low) was counterbalanced. The tone was followed by an blank interval of 500 ms. Depending on the between-subject variable *Group*, the auditory cue was at times followed by a feedback message (“TOO SLOW”) that was shown on screen for 2 s. The Procedure section provides more information concerning the purpose of this message. Next, a blank interval of 500 ms occurred.

Threat trials were marked by a chance of 1 out of 11 that there would be actual electrocutaneous stimulation. These trials are referred to in the remainder of the manuscript as ‘pain trials’. Participants were not informed of this proportion. In case of a pain trial, no other stimuli were presented but the electrocutaneous stimulus. In the remaining 10 threat trials, as well as in all neutral trials (10 in number), the auditory cue was followed by the administration of the SJ stimuli by the tactors on both hands. The stimuli were separated in time by 1 of 10 possible stimulus onset asynchronies (SOAs; -120, -60, -30, -15, -5, +5, +15, +30, +60 or +120 ms. These SOAs have also been used in multiple TOJ experiments [8,17]. In SJ experiments, it is customary to code SOAs so that negative values indicate that the test stimulus was presented first. In this study, we will regard stimuli at the threatened side as test stimuli, while stimuli at the opposite side will be labeled as reference stimuli. In the remainder of the manuscript, negative SOAs thus refer to trials in which the stimulus at the pain location preceded the stimulus at the pain-irrelevant location. Every SOA occurred an equal number of times during the course of the experiment (5 times per block, per condition).

Participants were asked to report aloud on which hand they felt the first tactile stimulus, by saying ‘left’ or ‘right’. If they did not sense a difference, they were asked to report this by saying ‘simultaneous’. When a painful stimulus replaced a SJ trial, participants were asked to report the hand on which this stimulus was felt. They had up to 5000 ms to respond before their response was coded as a blank. The experimenter coded all responses using the laptop keyboard.

### Procedure

Participants were given a brief description of the experiment and asked to fill in the informed consent form. They then completed a custom-made pre-test questionnaire, which is described in the self-report measures section below. Tactors and electrodes were then attached to the locations described above. Because it has been shown that somatosensory sensitivity can vary depending on which location of the body is stimulated [18], we first obtained appropriate tactile stimulation amplitudes for each hand. Our goal was to ensure that participants perceived tactile stimulation equally intense on both hands hand, so as not to give an advantage to either side. Our custom-made adaptive procedure, based on the double random staircase procedure, was designed as follows.

Participants were first given an orientation stimulus at 60 percent of the maximum capacity (and thus with a power of 0.612 watts) on the left hand. One second after that, a tactile stimulus was administered to the right hand. The amplitude of this second stimulus was taken from one of two staircases, which were alternated randomly for an equal number of times in total. The starting value for the first staircase was a random integer between 55 and 59, while the starting value of the second staircase was a random integer between 61 and 65. This way we ensured that participants would encounter both a stimulus that was higher in actual amplitude, and one that was lower in amplitude. After each pair of stimuli, participants were asked whether they perceived the second stimulus as “a lot stronger”, “stronger”, “equally strong”, “weaker” or “a lot weaker”. Their response determined the next value in the staircase (respectively 5 units down, 1 unit down, no change, 1 unit up or 5 units up). This procedure ran for 16 repetitions. The continuous coupling of orientation stimuli and to-be-rated stimuli was intended to ensure participants could adequately compare both sensations, making sure there was no gradual shift in memory of how the stimulus was perceived. It also served to prevent divergent sensitization effects on both hands. An average was made of all amplitude values that participants had reported to perceive equally strong. This value was used in the main experiment (Table 1).

**Table 1:** Stimulus levels.

Participants	Tactile intensity (left) (W)	Tactile intensity (right) (W)	Electrocutaneous intensity (left) (mA)	Electrocutaneous intensity (right) (mA)
1	0,61	0,53	1,40	1,50
2	0,61	0,51	1,70	2,50
3	0,61	0,50	1,50	1,50
4	0,61	0,56	1,40	1,50
5	0,61	0,65	1,70	2,10
6	0,61	0,60	2,50	2,40
7	0,61	0,57	2,40	2,00
8	0,61	0,63	3,30	3,90
9	0,61	0,42	2,80	2,70
10	0,61	0,54	3,00	3,00
11	0,61	0,40	3,00	3,00
12	0,61	0,52	2,50	2,80
13	0,61	0,44	2,60	2,50
14	0,61	0,74	3,50	4,00
15	0,61	0,70	3,40	3,70
16	0,61	0,36	2,70	2,40
17	0,61	0,44	2,70	3,10
18	0,61	0,60	2,10	2,10
19	0,61	0,58	3,50	4,30
20	0,61	0,41	3,50	3,80
21	0,61	0,52	3,50	3,90
22	0,61	0,81	2,40	3,50
23	0,61	0,64	2,40	2,30
24	0,61	0,63	3,80	3,80
25	0,61	0,59	3,20	3,50
26	0,61	0,66	3,70	3,50
27	0,61	0,57	3,60	2,90
28	0,61	0,64	2,90	2,70
29	0,61	0,50	3,90	3,00
30	0,61	0,57	3,40	3,70
31	0,61	0,53	2,70	2,40
32	0,61	0,61	3,80	3,60
33	0,61	0,66	4,00	3,20
34	0,61	0,56	4,40	4,70
35	0,61	0,46	3,80	3,40
36	0,61	0,65	4,40	5,00
37	0,61	0,44	3,20	3,60
38	0,61	0,70	3,60	3,60
39	0,61	0,79	4,20	4,90
40	0,61	0,44	3,30	3,40
M	0,61	0,57	3,04	3,14
SD	0,00	0,11	0,81	0,88

An overview of final stimulus intensity levels for tactile and electrocutaneous stimulation, following adaptation procedures, as well as their mean value (M) and standard deviation (SD).

In the following preparatory phase, we determined amplitudes for the electrocutaneous stimulation. We did this for each hand separately, using a double random staircase procedure of 14 steps. In this procedure, one staircase started with a value chosen between 15 and 19 (respectively 1.5 mA and 1.9 mA), whereas the other staircases started with a value between 20 and 24 (respectively 2.0 mA and 2.4 mA). Participants were asked to rate each stimulus on an 11-point scale (0 = “no pain”, 10 = “unbearable pain”). Reponses determined the next value in the corresponding staircase: a rating over 7 meant 1 unit down, a rating of 7 meant no change, and a rating under 7 meant 1 unit up. We took the average of all values to which participants gave a pain intensity rating of 7. This way we obtained pain intensities for both hands (Table 1), which we then used in the further course of the experiment.

We proceeded by introducing the participants with the SJ paradigm and explained the nature of the task. We presented them with 22 practice trials. Every SOA was presented twice, with two additional pain trials intermixed. We only proceeded when participants scored 100% accuracy on the trials with the largest SOA (+/- 120 ms). Next, we informed participants about the meaning of the auditory cues. Dependent on which group they were placed in (between-subjects variable of GROUP), participants received additional instructions with regard to the use of the foot pedal. In the **pain control group** (20 participants), participants were instructed that they could significantly reduce the chance of receiving painful stimuli throughout the experiment, by pressing down on the pedal as soon as they heard the threat-signaling cue. In reality, the timing and occurrence of painful stimuli were predetermined, ensuring that participants in the pain control group received an equal amount of pain stimuli as those in the comparison group. In other words, our goal manipulation depended on subjective control, rather than actual control. In this group, the feedback message was shown only during pain trials. This was done in an effort to reinforce participants’ perception of contingency between their (failed) pain control behavior and the occurrence of electrocutaneous stimuli.

In the **comparison group** (20 participants), participants were also instructed to press down on the pedal upon hearing the threat-signaling cue. These participants, however, were told this served to obtain additional measures of attention and concentration. No instructions related to pain control were given whatsoever. These participants were shown the feedback message at random times throughout the experiment. As such, they were not contingent with the presentation of painful stimulation. Still, the message strictly followed threat cues, as this was the only trial type in which these participants were requested to execute the timed button press. Four SJ blocks were then presented, as described above. The presentation order was counterbalanced with regard to modality and threat location.

### Self-report measures

Prior to the experiment, participants filled in a custom-made questionnaire, gauging for pre-existing pain-related conditions and episodes. All ratings (e.g., “To what degree were you unable to conduct daily activities during the past six months because of your pain?”) were indicated on an 11-point Likert scale. In addition, each experimental block was followed by a quick questionnaire, gauging for effort (“To what extent did you put effort into the task?”), concentration (“How well could you concentrate on the task?”), attention (“How much attention did you pay to the somatosensory stimuli?”; “How much attention did you pay to the electrocutaneous stimuli?”), fear related to either cue (“To what extent did you fear that a high/low tone would be followed by an electrocutaneous stimulus?”), pain expectancy related to either cue (“To what extent did you expect an electrocutaneous stimulus to follow the high/low tone?”), pain perception (“How painful did you find the electrocutaneous stimulus?”), anxiety (“How anxious did you feel during this block?”) and fatigue (“How tiresome did you find this block?”). All questions were answered on an appropriately anchored 11-point Likert scale. Answers were averaged over blocks per participants, prior to analysis. Finally, upon completion of all experimental blocks, participants completed the Pain Catastrophizing Scale (PCS) [19,20].

### Statistical analyses

**Fig 1:**
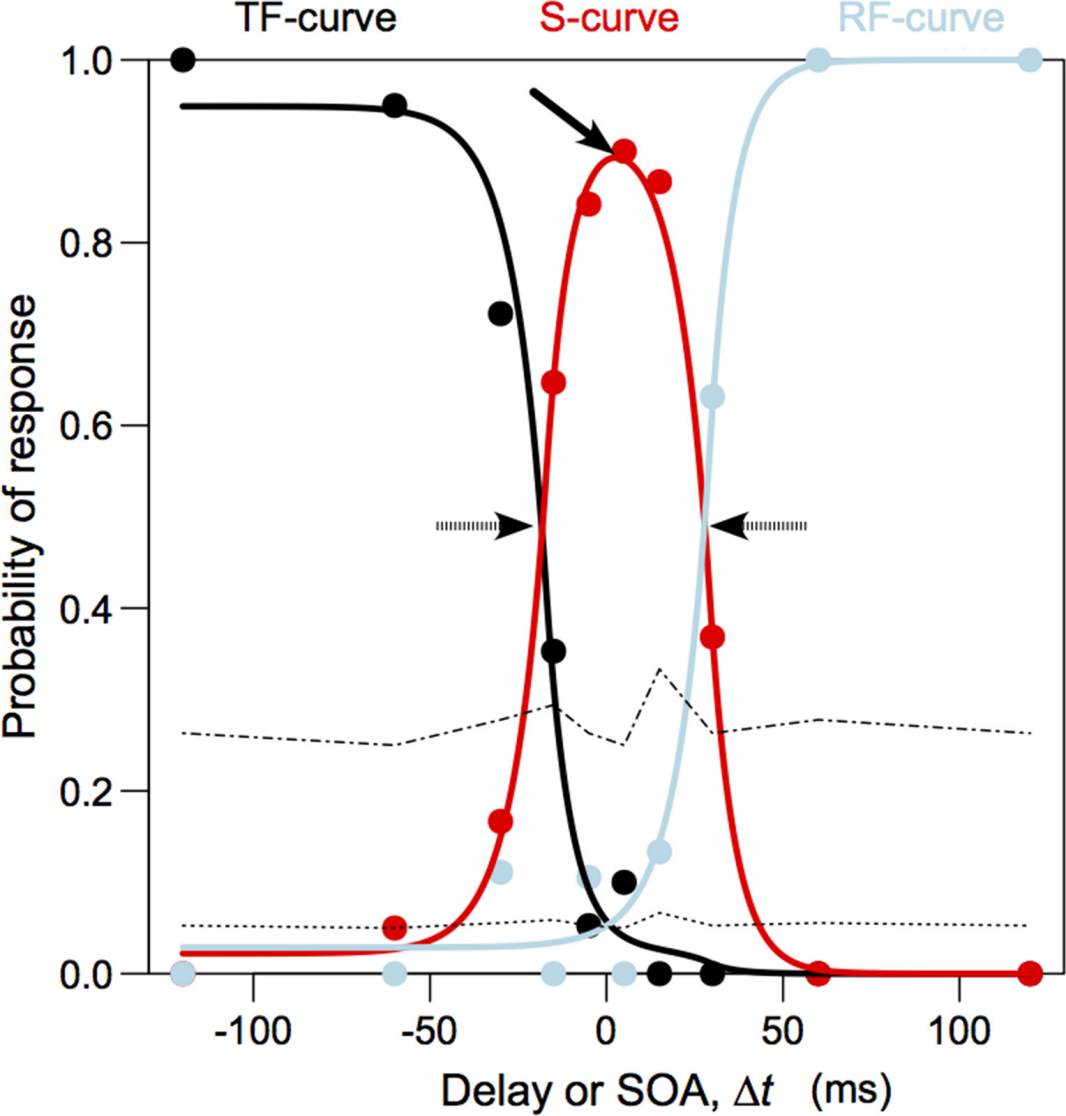
Performance measures in ternary synchrony judgment (SJ3) paradigms. Caption: We derived performance measures from the model that is fitted to the data points (solid dots). Peak PSS is defined as the peak of the S-curve (solid arrow), while midpoint PSS is defined as the midpoint between the intersections of the TF (test stimulus first)- and S (synchronous)-curve, and the RF (reference stimulus first)- and S-curve, respectively (striped arrows). This figure was made using the R-package described in [22] and slightly adapted.

Participants not reaching a mean accuracy of 80% on trials with the largest SOAs (+/- 120) were excluded from further analyses [8,21]. We then analyzed performance on the SJ-task by fitting these data to functions based on an independent-channels model, as described in [22] (Fig 1). In this instance, three-curves were fit to the data of each condition, for every participant. The proportions of responses per SOA that indicate that a participant first perceived the stimulus at the pain location (TF: test stimulus first) were approximated with a first curve. Those indicating that the stimulus presented on the opposite hand was perceived first (RF: reference stimulus first) were approximated by a second curve. Finally, simultaneous (S) responses were fitted with a third curve. There are two possible ways to define the PSS in an SJ experiment. It can either be calculated as the SOA that is associated with the maximal chance of receiving a ‘simultaneous’ response (the **peak** of the S-curve), or as the **midpoint** between two boundaries (the point in between the intersections of the S-curve with either the TF-curve, or the RF-curve). If judgments are perfectly symmetrical, these points should coincide. Empirically speaking, this is rarely the case [11,23]. In this study, both measurements will therefore be examined.

Using these fits, we obtained PSS measures for each condition. Our coding scheme is such that the sign of our PSS measurements indicates the direction of potential attentional shifts. Positive values indicate that stimuli stemming from the threatened location should be presented *after* stimuli originating on the opposing hand for both to be perceived as simultaneously occurring. Negative values refer to the opposite. Correspondingly, positive PSS measurements indicate an attentional shift towards the pain location, whereas negative values suggest attention to be drawn away from it.

Participants with PSS-values greater than the largest SOA were removed from the dataset [24]. PSS values were analyzed using a mixed-effects model with Gaussian link function, as implemented in the R package ‘lme4’ [25]. The statistical modeling procedure was as follows. First, all relevant factors and their interactions were entered in the model as fixed factors. These included *Threat* (threat trials vs. neutral trials) and *Group* (pain control group versus comparison group). By default, a random effect was added introducing adjustments to the intercept conditional on each subject separately. Next, we determined whether the addition of random effects was necessary for the within-subject fixed factor of *Threat*. If a random effect increased the model’s goodness of fit, we included it in the final model. In a second step, we sought out the most parsimonious model that fit the data by restricting the full model systematically, starting with higher-order terms. All model comparisons were made using likelihood-ratio tests. In a third and final step, we inspected the ANOVA table of the final model and tested specific hypotheses about possible main effects or interactions (see [6,26,27], for a similar approach). All contrast analyses were corrected for multiple testing according to the corrections of Holm-Bonferroni [28].

As discussed in the introduction, we hypothesized that the anticipation of a painful stimulus would induce attentional prioritization of the threatened location (hypothesis 1). In addition, we expected that this prioritization would depend on the *Group* factor. More specifically, we expected to see either more pronounced effects in the pain control group (hypothesis 2a: *Threat* x *Group* interaction), generalized effects across threat and neutral trials (hypothesis 2b: *Group* main effect), or a combination of both.

## Results

### SJ data

We eliminated 4 participants (all of which in the comparison group) whose accuracy on trials with the largest SOA (± 120 ms) fell under the cut-off level of 80 percent. None of the remaining participants showed PSS values outside of the SOA range. One participant’s data did not yield a model suitable to obtain a midpoint PSS measurement, due to a noticeable lack of S-responses. This participant was omitted from further analyses (pain control group).

**Fig 2:**
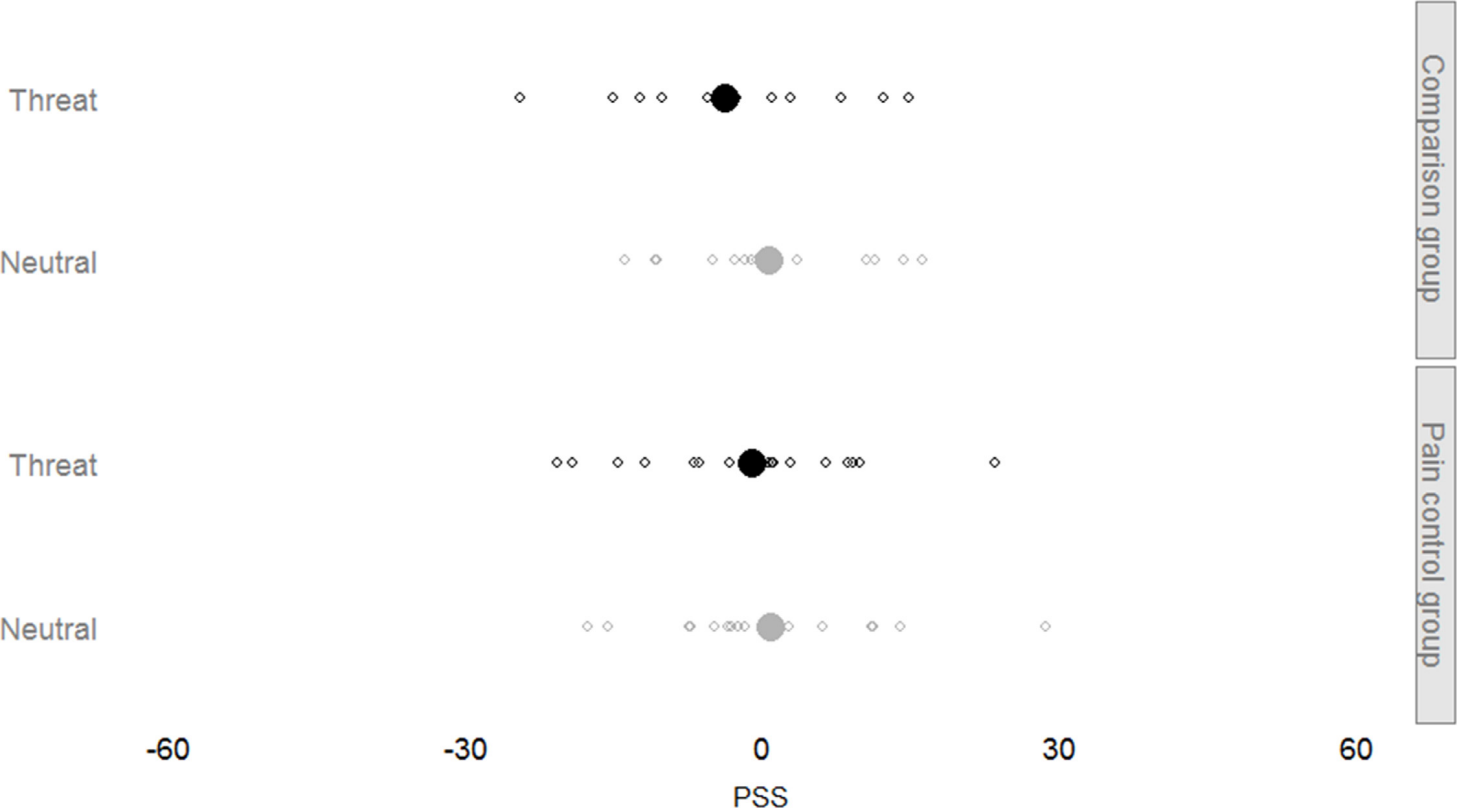
Peak PSS overview. We compared individual Point of Subjective Simultaneity (PSS) measurements across conditions. For every participant in both the comparison group and the pain control group, we calculated the peak PSS (smaller, hollow circles). Mean PSS values are indicated as well (larger, solid circles). PSS values based on threat trials are black, whereas PSS values based on neutral trials are grey. More positive values means somatosensory information from the threatened body location is processed quicker than somatosensory information from the other hand. The illustrated pattern shows no clear bias towards either stimulus, neither due to the threat manipulation or the goal manipulation.

**Fig 3:**
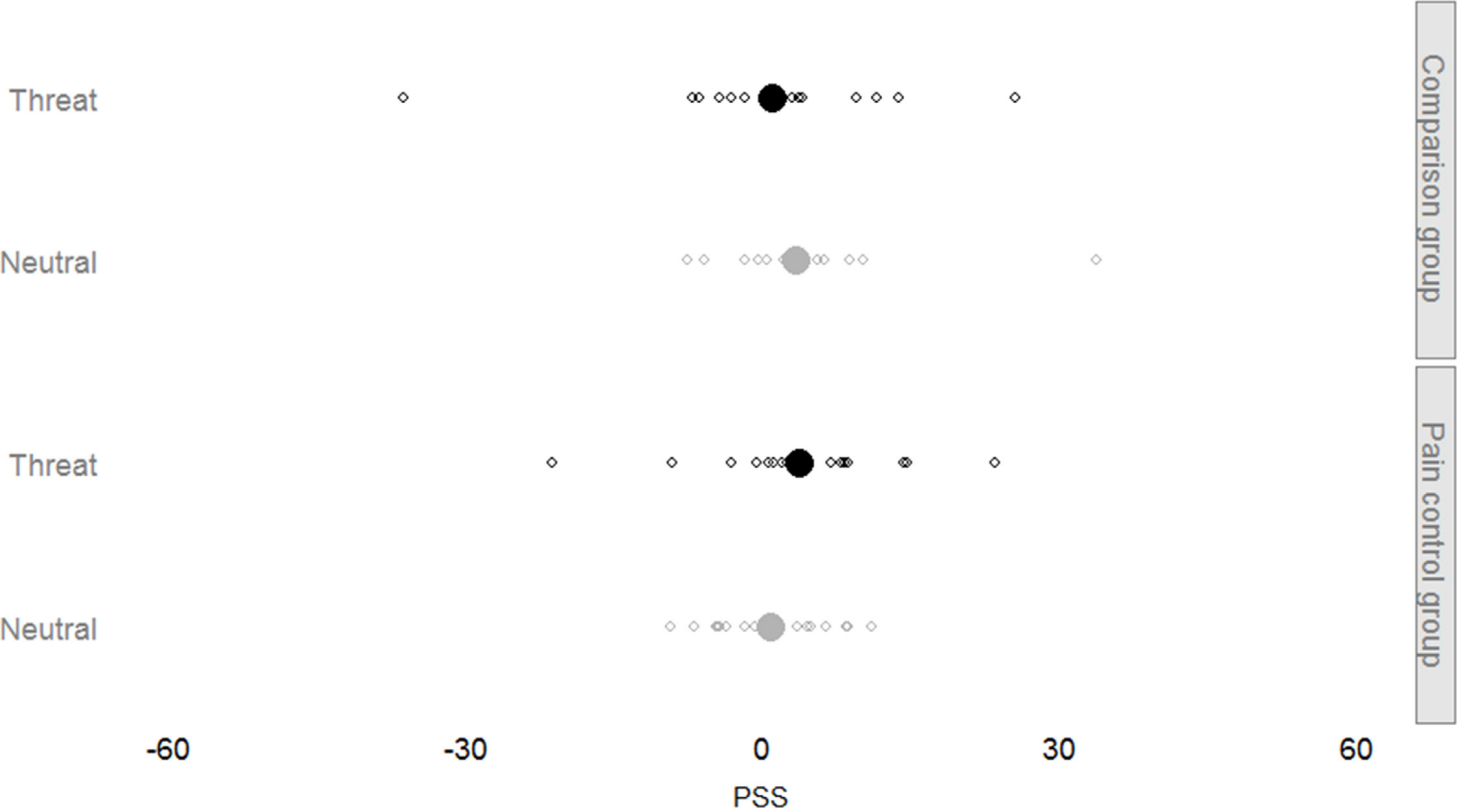
Midpoint PSS overview. Similar to the procedure for peak PSS values, we also compared PSS values using midpoint measurements. Again, no distinctive biases can be demonstrated using these measurements.

We first fitted a model containing all fixed factors and interactions and a random subject-based intercept to both the PSS peak data and the PSS midpoint data (see Figs 2 and 3 for an overview). No additional random effects were necessary in either model. The final models yielded no significance for any of the included factors (*Threat* and *Group*) or their interaction (all *p* > .05). Removing the interaction term from these models did not render any of the fixed factors significant.

### Self-report data and manipulation check

Participants assessed their own health as ‘very good’, on average. One participant reported to be revalidating from a serious injury, but did not report any pain at the time of testing. One participant reported to suffer from heart disease. There was no reason to suspect this influenced our data. No other participants reported any serious medical conditions, nor did anyone report to suffer from mental illness of any sort.

Twenty-four participants had experienced some form of pain during the past 6 months (M = 25.94 days, SD = 36.92 days). This pain had an average intensity rating of 4.63 (SD = 1.53) and an average disability rating of 2.92 (SD = 2.38). One of these reported to have suffered from her pain complaint for 180 days (intensity rating: 7; disability rating: 7). We found no evidence that these participants significantly distorted the data. One participant reported having taken a pain killer earlier during the day. However, this participant underwent the same pain calibration procedure, and reported perceiving the electrocutaneous stimulation as painful over the course of the experiment. Twelve participants reported feeling pain at the moment of testing, on a Likert-scale ranging from “no pain” to “worst possible pain”. Their average pain intensity ratings were low (M = 1.69, SD = 0.95.

PCS scores were not significantly different between groups (comparison group: M = 10.75, SD = 8.95; pain control group: M = 11.05, SD = 8.59) (*χ*^*2*^ = 0.01, *p* = .91). Over the course of the experiment, the electrocutaneous stimulus was rated moderately painful in both groups (comparison: M = 4.73, SD = 1.68; pain control: M = 3.83, SD = 2.42) with no significant difference between them (*χ*^*2*^ = 1.86, *p* = .17). To verify the effect of the threat manipulation, we applied an ANOVA with the factors CUE (threatening versus neutral) and GROUP (comparison versus pain control) on fear and pain expectancy ratings. With regard to fear ratings, we found a main effect of the CUE variable (*χ*^*2*^ = 78.26, *p* < .001), indicating that participants felt more fearful upon hearing the threat cue (M = 4.88, SD = 2.10) compared to the neutral cue (M = 1.11, SD = 1.65) (d = 2.00, 95% CI = 1.45–2.56). This confirmed that the threat manipulation was successful. A comparable pattern was found with respect to pain expectancy ratings, again showing a significant main effect of CUE (*χ*^*2*^ = 54.24, *p* < .001). Similarly, hearing the threatening cue led to more pain expectancy (M = 4.39, SD = 2.30) compared to hearing the neutral cue (M = 1.09, SD = 1.60) (d = 1.67, 95% CI = 1.14–2.19). No other group differences were found (Table 2).

**Table 2:** Self report data.

	M com	SD com	M cont	SD cont	*χ²*	*p*
Pain experience	4.73	1.68	3.83	2.42	1.86	.17
Anxiety	3.84	2.19	3.10	2.59	0.95	.33
Attention to painful stimuli	4.56	2.17	3.88	2.14	1.02	.31
Attention to tactile stimuli	7.40	2.24	6.38	2.33	2.01	.16
Concentration	7.38	1.67	7.29	0.93	0.04	.84
Effort	7.85	1.51	7.64	0.95	0.28	.59
Fatigue	5.29	2.34	5.11	1.89	0.07	.79
Fear (neutral cue)	1.00	1.73	1.21	1.61	0.06	.81
Fear (threat cue)	4.96	2.12	4.80	2.13	0.16	.69
Pain expectancy (neutral cue)	1.05	1.70	1.13	1.54	< 0.01	.93
Pain expectancy (threat cue)	4.43	2.12	4.36	2.52	0.02	.88
PCS	10.75	8.95	11.05	8.59	0.01	.91

This table shows mean values and standard deviations for self-report ratings given by the comparison group (com) and the pain control group (cont), as well as their comparison using univariate ANOVAs

## Discussion

When an individual is under threat of pain, he or she tends to devote more attention towards the threatened location of the body [5,6,8]. In addition, attempts to avoid this pain have been shown to lead to more generalized effects, expanding this prioritization to neutral trials [6]. In the present study, we sought to provide additional support for these findings using a SJ3 paradigm. Our data failed to support the existence of the hypothesized prioritization effects. We found no evidence that the threat of pain led to faster processing of stimuli at the endangered body site, nor were we able to show that such effects could be modulated by pain control attempts.

There are several possible explanations for this lack of significant results. First, it is possible that the effects documented in previous judgment paradigms [7,8,10] are caused by mechanisms different to the proposed attentional shift explanation. For instance, TOJ paradigms have been suggested to be particularly susceptible to response bias. Applied to these studies, this could mean that participants will resort to naming the threatened location when they are unsure of the order in which they perceived the experimental stimuli. In other words, the effect of anticipated pain could manifest itself in the decision making process, rather than the actual sensory processing [13].

In this regard, SJ experiments are thought to hold the advantage, as the tertiary response option can eliminate response bias [11]. Our inability to reproduce results could then be seen as an indication that such response bias was a crucial–or even, sole–determinant of earlier results. Still, there are arguments against this notion. Recent studies demonstrated spatial prioritization of a threatened location by means of a tactile change detection (TCD) paradigm [6,29]. In a TCD experiment, participants are requested to judge whether or not they sensed a change in subsequent patterns of tactile stimulation. The derived data can then be analyzed to see if change detection is dependent on the crucial involvement of particular locations, such as a body location where pain is expected to occur. It is evident that the response format in TCD (“change” or “no change”) is orthogonal to the variable indicating the involvement of ‘special’ locations. Consequently, such design leaves no room for response bias–of which aforementioned TOJ experiments are suspect–to take place. In spite of this, these TCD studies have all shown evidence of threat-induced spatial prioritization, using an arguably more complex experimental setup. As such, these results suggest that the findings from TOJ experiments can be interpreted as support for the manifestation of spatial prioritization, as opposed to a mere reflection of response bias.

A second explanation for our underwhelming results is implied in the distribution of the obtained data. Closer inspection to the amount of TF-, S- and RF-responses reveals that subjects greatly differ with regard to the standard they use to decide which response option they should choose. For example, some participants report that they perceived the stimuli to occur simultaneously less than 8 times out of an approximate total of 400 trials (not withholding trials where the response time limit was exceeded), while others resort to such S-responses over 190 times throughout the experiment. These differences make extraction of useful measures of perception a difficult task. This problem has been documented before, particularly with regard to the ternary SJ-format [12]. It has no clear-cut remedy, other than returning to a TOJ format and making appropriate adjustments there–such as the implementation of an orthogonal design.

Third, it may be important to note that the use of feedback messages–pertaining to the effectiveness of pain avoidance attempts–could have had an inadvertent effect. In the pain control group, specifically, this message was contingent with the administration of an electrocutaneous stimulus. When a participant went through a pain trial–that is, a threat trial resulting in actual pain–this message was intended to further encourage control attempts and to reaffirm their necessity. However, due to design, participants could have been aware that they were ‘safe’ when they did not see a visual feedback message but felt the tactile stimuli instead. If somatosensory monitoring is at the core of spatial prioritization towards a threatened location, this could have interfered with the attentional effects we meant to investigate. Indeed, the feedback message–or lack thereof–in our experiment may have prematurely indicated that the pain location was in fact no longer threatened, eliminating the need to dedicate a surplus of attentional resources towards it. This rationale only applies to the pain control group, given that feedback in the comparison group was not contingent on the actual manifestation of pain. Moreover, we feel that this explanatory avenue would be more likely if we had implemented positive feedback when pain was ‘successfully avoided’. Still, investigating the potential role of prioritization as a monitoring mechanism may be an interesting direction for future research.

Fourth, it may be worthwhile highlighting that we fit our data with psychometric curves based on the independent-channels model [11,21], as opposed to Gaussian or logistic functions [8,14,24,30]. It has been argued that the former approach holds the advantage here on multiple fronts. For example, all the parameters that are estimated in the fitting process are of theoretical significance, and therefore interpretable (e.g. observer misreports). When arbitrary functions are used instead, this is not the case (e.g. σ and μ in Gaussian curves). Additionally, the independent-channels model is able to accommodate asymmetries and irregularities which are typically present in the data of an individual observer–a feature lacking in Gaussian or logistic curves. However, in spite of these arguments, it is unlikely that this difference in fitting strategy is solely responsible for the inconsistency our results show with existing literature.

Fifth, with respect to the specific research question we aimed to answer in this study–that is, examining the effect of a sustained focus of attention to a threatened body location–we deliberately chose to keep the pain location constant. The auditory cue we used to indicate possible administration of pain was presented to both ears simultaneously, and thus was not linked to either location. An alternative approach could be to investigate the effects of threat-induced transient shifts of attention to different body locations, for example by cueing the expected pain location on a trial-to-trial basis [31] or by experimentally controlling the location of threatening cues [32].

Finally, there is a possibility that the discrepancy between this study and its predecessor TOJ studies is not caused by its distinct methodology, but is rather due to individual differences. As such, an interesting avenue for future research would be to submit participants to parallel experiment sessions in which both paradigms are contrasted, preferably in a counterbalanced order. This would allow us to directly compare the psychometrics of both tasks on an individual level, thus potentially eliminating this explanation.

## Conclusion

In conclusion, this study failed to corroborate effects of pain-related attentional prioritization, even though these effects are supported by the results of multiple contemporary studies. This inability, given the arguments we have listed in favor of our current approach, invites critical reflection on predominant methodology.

## Supporting Information

S1 Dataset Complete dataset. This dataset includes pretest questionnaire data, posttest questionnaire data, and experimental (SJ3) data.
(ZIP)
